# Correlation Between Middle Cerebral Artery Peak Systolic Velocity and Neonatal Haemoglobin as a Marker of Fetal Anaemia

**DOI:** 10.7759/cureus.109456

**Published:** 2026-05-22

**Authors:** Ravindra S Pukale, Manikonda B SatyaSri Alekhya

**Affiliations:** 1 Obstetrics and Gynaecology, Adichunchanagiri Institute of Medical Sciences, Adichunchanagiri University, BG Nagara, IND

**Keywords:** anemia at birth, cord blood hemoglobin, doppler ultrasonography, fetal anemia, mca-psv

## Abstract

Introduction: Fetal anaemia is an important cause of perinatal morbidity and mortality. Middle cerebral artery peak systolic velocity (MCA-PSV) measured by Doppler ultrasound has emerged as a reliable non-invasive tool for its assessment. However, its correlation with haemoglobin levels in heterogeneous clinical populations requires further evaluation. This study aimed to evaluate the correlation between MCA-PSV and cord blood haemoglobin and to assess the diagnostic utility of MCA-PSV in predicting anaemia at birth.

Materials and methods: This prospective observational study was conducted over six months at a tertiary care centre and included 100 singleton pregnancies. MCA-PSV was measured using standardised Doppler techniques and expressed as multiples of the median (MoM). Cord blood haemoglobin was measured within 24 hours of birth. Correlation between MCA-PSV and haemoglobin was assessed using Pearson’s correlation coefficient. Diagnostic performance of MCA-PSV MoM ≥1.5 was evaluated using sensitivity, specificity, and predictive values.

Results: A significant moderate inverse correlation was observed between MCA-PSV and cord blood haemoglobin (r = −0.42, p < 0.001). Anaemia at birth was present in 86 (86%) cases, with moderate-to-severe anaemia in 52 (52%). MCA-PSV and MoM values were significantly higher in anemic neonates (p < 0.001). MCA-PSV MoM increased with the severity of anaemia (p < 0.001). A threshold of ≥1.5 MoM showed excellent specificity (100%) and positive predictive value (100%) but low sensitivity (8.1%).

Conclusion: MCA-PSV demonstrates a significant inverse correlation with haemoglobin levels and is a useful non-invasive marker for fetal anaemia. While highly specific, its low sensitivity limits its role as a screening tool, emphasising the need for integrated clinical assessment.

## Introduction

Fetal anaemia is a significant contributor to perinatal morbidity and mortality, particularly when severe or left undiagnosed [1]. It may result from a variety of conditions, including red blood cell alloimmunisation, intrauterine infections, haemoglobinopathies, fetomaternal haemorrhage, and complications of multiple gestations [2]. Traditionally, diagnosis relied on invasive procedures such as amniocentesis and cordocentesis for direct estimation of fetal haemoglobin [3]. Although these methods are accurate, they are associated with risks such as fetal loss, infection, and preterm labour, thereby necessitating the development of safer, non-invasive alternatives [3].

Advances in obstetric ultrasonography, particularly Doppler velocimetry, have revolutionised fetal assessment by enabling real-time evaluation of fetal circulation [4,5]. Among various Doppler parameters, the middle cerebral artery (MCA) has emerged as a key vessel for assessment due to its accessibility and sensitivity to haemodynamic changes [6]. In the setting of fetal anaemia, reduced blood viscosity and compensatory mechanisms lead to increased cerebral blood flow, which manifests as elevated peak systolic velocity (PSV) in the MCA [7,8].

The physiological basis of this phenomenon lies in the fetal adaptive response to decreased oxygen-carrying capacity [9]. As haemoglobin levels decline, cerebral vasodilation occurs to preserve oxygen delivery to vital organs, particularly the brain [9,10]. This results in increased flow velocity within the MCA, which can be detected non-invasively using Doppler ultrasound [10]. Numerous studies have demonstrated a strong inverse relationship between MCA-PSV and fetal haemoglobin levels, establishing its role as a reliable indicator of fetal anaemia [11-13].

MCA-PSV values expressed as multiples of the median (MoM) for gestational age have been widely used in clinical practice, with values ≥1.5 MoM considered predictive of moderate-to-severe fetal anaemia [14,15]. This non-invasive approach has largely replaced invasive testing for initial screening and follow-up in high-risk pregnancies, allowing for timely intervention and improved perinatal outcomes [16]. In this context, the present study aimed to evaluate the correlation between middle cerebral artery peak systolic velocity and cord blood haemoglobin, reflecting fetal anaemia, and to assess its diagnostic utility in predicting anaemia at birth.

## Materials and methods

This prospective observational study was conducted in the Department of Obstetrics and Gynaecology at Adichunchanagiri Institute of Medical Sciences over a period of six months from October 2025 to March 2026, after obtaining approval from the Institutional Ethics Committee (IEC No.: AIMS/IEC/256/2025). The study included pregnant women admitted during the study period who met predefined eligibility criteria.

Pregnant women with singleton gestations undergoing fetal Doppler assessment and having documented MCA-PSV measurements were included. Only those cases in which cord blood haemoglobin estimation was available within 24 hours of birth were considered. Pregnancies complicated by multiple gestation, major congenital anomalies, chromosomal abnormalities, or poor-quality/non-standard Doppler recordings were excluded to maintain data reliability.

Sample size was calculated for correlation analysis using Fisher’s Z transformation, based on prior evidence demonstrating a strong inverse correlation between MCA-PSV and fetal haemoglobin, as reported by Slaghekke et al. (r = −0.86) [17]. Using the formula

$n = \frac{(Z_{\alpha} + Z_{\beta})^2}{\left(0.5 \times \ln\left(\frac{1 + r}{1 - r}\right)\right)^2} + 3$

with a 95% confidence level (Zα = 1.96) and 80% power (Zβ = 0.84), the minimum required sample size was calculated to be approximately 15. Although the calculated minimum sample size was 15, a larger sample size of 100 was included to enhance statistical reliability and improve the precision of estimates.

Fetal MCA-PSV measurements were obtained as part of routine obstetric Doppler ultrasonography using standardised techniques, with the Doppler sample volume placed in the proximal third of the middle cerebral artery and appropriate angle correction applied. The values were expressed both in absolute terms (cm/s) and as multiples of the median (MoM) for gestational age [18]. Cord blood haemoglobin levels were measured from venous blood samples collected within 24 hours of delivery as part of standard neonatal evaluation. Anaemia at birth was defined based on cord blood haemoglobin levels and graded as mild (10-13.9 g/dL), moderate (7-9.9 g/dL), and severe (<7 g/dL) [19].

Relevant maternal, fetal, and neonatal data, including gestational age, gravidity, fetal growth status, Doppler indices, birth weight, NICU admission, and need for postnatal intervention, were recorded in a structured data collection proforma. Postnatal intervention was defined as any therapeutic measure, such as packed red blood cell transfusion, exchange transfusion, phototherapy, or intravenous immunoglobulin, administered based on neonatal clinical status. All data were anonymised and assigned unique identifiers to ensure confidentiality.

Data were entered into Microsoft Excel (Microsoft Corporation, Redmond, Washington) and analysed using IBM SPSS Statistics for Windows, Version 26 (Released 2019; IBM Corp., Armonk, New York). Continuous variables were expressed as mean ± standard deviation, while categorical variables were presented as frequencies and percentages. The relationship between MCA-PSV (both absolute and MoM values) and cord blood haemoglobin was assessed using Pearson’s correlation coefficient. Comparisons between groups were performed using an independent sample t-test or one-way ANOVA, as appropriate, while categorical variables were analysed using the chi-square test. Diagnostic performance of MCA-PSV MoM ≥1.5 in predicting anaemia at birth was evaluated using sensitivity, specificity, positive predictive value, and negative predictive value [18]. A p-value of <0.05 was considered statistically significant.

## Results

The cohort had a mean maternal age of 26.9 years and a mean gestational age of 33 weeks. Nearly one-third of fetuses had SGA/FGR (small for gestational age/fetal growth restriction), accounting for 29 (29%) cases, while Doppler indices remained largely within the normal range (Table 1). 

**Table 1:** Baseline Maternal, Fetal, and Doppler Characteristics (n = 100) Data are presented as mean ± standard deviation (SD) or number (%). SGA/FGR: small for gestational age/fetal growth restriction; MCA-PSV: middle cerebral artery peak systolic velocity; MoM: multiples of the median; PI: pulsatility index. Descriptive statistics were applied.

Variable	Category	Value
Maternal age (years)	Mean ± SD	26.9 ± 4.8
Gestational age at Doppler (weeks)	Mean ± SD	33.1 ± 4.2
Gravidity	Primigravida	46 (46%)
	Multigravida	54 (54%)
MCA-PSV (cm/s)	Mean ± SD	52.7 ± 10.4
MCA-PSV (MoM)	Mean ± SD	1.17 ± 0.23
MCA Pulsatility Index	Mean ± SD	1.02 ± 0.12
Estimated fetal weight (g)	Mean ± SD	2090 ± 710
Fetal growth status	SGA / FGR	29 (29%)
	Appropriate for gestational age	71 (71%)

Anaemia at birth was highly prevalent, occurring in 86 (86%) cases, with a substantial proportion requiring NICU admission, accounting for 42 (42%) cases. Moderate-to-severe anaemia accounted for 52 (52%) of cases, indicating a significant disease burden (Table 2). 

**Table 2:** Neonatal Outcomes (n = 100) Data are presented as mean ± SD or number (%). The severity of anaemia was classified based on cord blood haemoglobin levels. Descriptive statistics were applied. NICU: neonatal intensive care unit.

Variable	Category	Value
Birth weight (g)	Mean ± SD	2111 ± 720
NICU admission	Yes	42 (42%)
	No	58 (58%)
Cord blood haemoglobin (g/dL)	Mean ± SD	13.5 ± 2.9
Anaemia at birth	Present	86 (86%)
	Absent	14 (14%)
Severity of anaemia	Mild (10–13.9 g/dL)	34 (34%)
	Moderate (7–9.9 g/dL)	33 (33%)
	Severe (<7 g/dL)	19 (19%)

MCA-PSV (both absolute and MoM values) showed a significant moderate negative correlation with cord blood haemoglobin (r = −0.42, p < 0.001), while MCA PI demonstrated a weak positive correlation (Table 3). 

**Table 3:** Correlation Between MCA Doppler Parameters and Cord Blood Haemoglobin Data are presented as Pearson’s correlation coefficient (r) with corresponding p-values. MCA-PSV: middle cerebral artery peak systolic velocity; MoM: multiples of median; PI: pulsatility index; Hb: haemoglobin. Pearson’s correlation test was applied.

Parameter	r	p-value	Interpretation
MCA-PSV (cm/s) vs Cord Hb	−0.42	<0.001	Moderate negative correlation
MCA-PSV (MoM) vs Cord Hb	−0.42	<0.001	Moderate negative correlation
MCA PI vs Cord Hb	+0.18	0.025	Weak positive correlation

Neonates with anaemia at birth had significantly higher MCA-PSV and MoM values and lower MCA PI compared to those without anaemia (p < 0.05 for all), indicating altered fetal hemodynamics (Table 4).

**Table 4:** Comparison of Doppler Parameters Between Anaemic and Non-anaemic Neonates Data are presented as mean ± SD. Independent Student’s t-test was applied for comparison between groups. A p-value of <0.05 was considered statistically significant. MCA-PSV: middle cerebral artery peak systolic velocity; MoM: multiples of median; PI: pulsatility index.

Parameter	Anaemia Present (n = 86)	Anaemia Absent (n = 14)	Test Statistic (t)	p-value
MCA-PSV (cm/s)	54.2 ± 10.1	44.3 ± 7.8	3.76	<0.001
MCA-PSV (MoM)	1.21 ± 0.22	0.96 ± 0.15	4.52	<0.001
MCA PI	0.99 ± 0.11	1.08 ± 0.10	−2.56	0.012

A significant increase in MCA-PSV MoM was observed with increasing severity of anaemia at birth (p < 0.001) (Table 5).

**Table 5:** MCA-PSV MoM According to Severity of Anaemia at Birth Data are presented as mean ± SD. One-way analysis of variance (ANOVA) test was applied. A p-value of <0.05 was considered statistically significant. MCA-PSV: middle cerebral artery peak systolic velocity; MoM: multiples of median.

Severity	MCA-PSV (MoM), Mean ± SD	Test Statistic (F)	p-value
Mild anaemia	1.10 ± 0.18	12.84	<0.001
Moderate anaemia	1.22 ± 0.20		
Severe anaemia	1.38 ± 0.25		

All fetuses with MCA-PSV MoM ≥1.5 had anaemia at birth, accounting for seven (100%) cases, although most anaemic cases fell below this threshold, indicating limited sensitivity (Table 6). 

**Table 6:** Association Between MCA-PSV MoM (≥1.5) and Anemia at Birth Data are presented as numbers (%). Chi-square (χ²) test was applied. A p-value of <0.05 was considered statistically significant. MCA-PSV: middle cerebral artery peak systolic velocity; MoM: multiples of median.

MCA-PSV MoM	Anaemia Present	Anaemia Absent	Total	Test Statistic (χ²)	p-value
<1.5	79 (84.9%)	14 (15.1%)	93 (100%)	10.21	0.001
≥1.5	7 (100%)	0 (0%)	7 (100%)		
Total	86 (86.0%)	14 (14.0%)	100 (100%)		

MCA-PSV MoM ≥1.5 showed excellent specificity (100%) but very low sensitivity (8.1%), supporting its role as a confirmatory rather than a screening tool for anaemia at birth (Table 7). 

**Table 7:** Diagnostic Performance of MCA-PSV MoM ≥1.5 Data are presented as percentages. Diagnostic accuracy analysis was applied. MCA-PSV: middle cerebral artery peak systolic velocity; MoM: multiples of median; PPV: positive predictive value; NPV: negative predictive value.

Parameter	Value
Sensitivity	8.1%
Specificity	100%
Positive predictive value (PPV)	100%
Negative predictive value (NPV)	15.1%

## Discussion

The present study demonstrated a significant inverse correlation between MCA-PSV and cord blood haemoglobin (r = −0.42, p < 0.001), supporting the established physiological relationship between increased cerebral blood flow velocity and reduced fetal haemoglobin levels. Similar findings have been reported by Teixeira et al., who observed a stronger inverse correlation (r = −0.69) in alloimmunised pregnancies, indicating that the strength of association may vary depending on the underlying aetiology [20]. The comparatively moderate correlation in the present study may be attributed to the inclusion of a heterogeneous population, including cases with fetal growth restriction and mixed risk factors, which can influence fetal haemodynamics.

The diagnostic performance of MCA-PSV MoM ≥1.5 in the present study showed excellent specificity (100%) but very low sensitivity (8.1%). In contrast, Scheier et al. reported high sensitivity in detecting severe fetal anaemia using similar thresholds, particularly in homogeneous populations with significant haemoglobin deficits [21]. This discrepancy can be explained by the higher proportion of mild and moderate anaemia at birth in the present study, where compensatory haemodynamic changes may not be sufficient to cross the 1.5 MoM cutoff. These findings suggest that MCA-PSV is more reliable as a confirmatory rather than a screening tool in mixed clinical settings.

The observed increase in MCA-PSV MoM with increasing severity of anaemia at birth in this study is consistent with the work of Mari et al., who demonstrated a strong linear inverse relationship between MCA-PSV and fetal haemoglobin levels [18]. Similarly, Delle Chiaie et al. reported good correlation and high diagnostic accuracy across different aetiologies of fetal anaemia [22]. However, the lower sensitivity observed in the present study compared to these reports likely reflects real-world clinical variability and inclusion of a broader spectrum of anaemia severity.

An important factor influencing the findings of this study is the presence of fetal growth restriction in a substantial proportion of cases. Turan et al. have shown that altered cerebral blood flow in growth-restricted fetuses may affect MCA Doppler indices independently of anaemia, thereby reducing diagnostic sensitivity [23]. Additionally, the weak correlation between MCA pulsatility index and haemoglobin observed in this study supports previous evidence that PSV is a more sensitive marker of fetal anaemia than resistance indices. Overall, while elevated MCA-PSV remains a highly specific indicator of fetal anaemia, its interpretation should be made in conjunction with the clinical context and associated risk factors to optimise diagnostic accuracy.

Strengths and limitations

The present study has several strengths, including its prospective design, standardised Doppler assessment of MCA-PSV, and use of cord blood haemoglobin as an objective reference for diagnosing anaemia at birth. The inclusion of both absolute MCA-PSV and MoM values, along with correlation, comparative, and diagnostic analyses, provides a comprehensive evaluation of its clinical utility. Additionally, the study reflects real-world clinical practice by including a heterogeneous high-risk population, thereby enhancing its external validity.

However, certain limitations must be acknowledged. The relatively small sample size and single-centre design may limit generalisability. The inclusion of mixed aetiologies, including fetal growth restriction, may have influenced MCA Doppler measurements and reduced diagnostic sensitivity. Furthermore, the low number of cases with MCA-PSV MoM ≥1.5 limits robust assessment of diagnostic performance, and the absence of longitudinal follow-up or stratification by aetiology may have constrained deeper analysis of disease-specific patterns.

## Conclusions

The present study demonstrates a significant inverse correlation between MCA-PSV and cord blood haemoglobin levels, reaffirming its role as a useful non-invasive marker for assessing fetal anaemia. Increasing MCA-PSV, particularly when expressed as multiples of the median, was associated with greater severity of anaemia at birth, supporting its physiological relevance. An MCA-PSV MoM threshold of ≥1.5 showed excellent specificity and positive predictive value, indicating its utility as a confirmatory tool; however, its low sensitivity limits its effectiveness as a standalone screening modality, especially in heterogeneous high-risk populations. These findings highlight that while MCA-PSV is valuable in the evaluation of fetal anaemia, its interpretation should be integrated with the clinical context and other parameters to optimise diagnostic accuracy and improve perinatal outcomes.
